# Genomic evolution of the globally disseminated multidrug-resistant *Klebsiella pneumoniae* clonal group 147

**DOI:** 10.1099/mgen.0.000737

**Published:** 2022-01-12

**Authors:** Carla Rodrigues, Siddhi Desai, Virginie Passet, Devarshi Gajjar, Sylvain Brisse

**Affiliations:** ^1^​ Biodiversity and Epidemiology of Bacterial Pathogens, Institut Pasteur, Université de Paris, Paris, France; ^2^​ Department of Microbiology and Biotechnology Centre, Faculty of Science, The Maharaja Sayajirao University of Baroda, Vadodara, Gujarat, India

**Keywords:** CRISPR/Cas system, genomic epidemiology, high-risk clone, IncF plasmid, NDM-5, pandrug resistance

## Abstract

The rapid emergence of multidrug-resistant *Klebsiella pneumoniae* is being driven largely by the spread of specific clonal groups (CGs). Of these, CG147 includes 7-gene multilocus sequence typing (MLST) sequence types (STs) ST147, ST273 and ST392. CG147 has caused nosocomial outbreaks across the world, but its global population dynamics remain unknown. Here, we report a pandrug-resistant ST147 clinical isolate from India (strain DJ) and define the evolution and global emergence of CG147. Antimicrobial-susceptibility testing following European Committee on Antimicrobial Susceptibility Testing (EUCAST) guidelines and genome sequencing (Illumina and Oxford Nanopore Technologies, Unicycler assembly) were performed on strain DJ. Additionally, we collated 217 publicly available CG147 genomes [National Center for Biotechnology Information (NCBI), May 2019]. CG147 evolution was inferred within a temporal phylogenetic framework (beast) based on a recombination-free sequence alignment (Roary/Gubbins). Comparative genomic analyses focused on resistance and virulence genes and other genetic elements (BIGSdb, Kleborate, PlasmidFinder, phaster, ICEfinder and CRISPRCasFinder). Strain DJ had a pandrug-resistance phenotype. Its genome comprised the chromosome, seven plasmids and one linear phage-plasmid. Four carbapenemase genes were detected: *bla*_NDM-5_ and two copies of *bla*_OXA-181_ in the chromosome, and a second copy of *bla*_NDM-5_ on an 84 kb IncFII plasmid. CG147 genomes carried a mean of 13 acquired resistance genes or mutations; 63 % carried a carbapenemase gene and 83 % harboured *bla*_CTX-M_. All CG147 genomes presented GyrA and ParC mutations and a common subtype I-E CRISPR-Cas system. ST392 and ST273 emerged in 2005 and 1995, respectively. ST147, the most represented phylogenetic branch, was itself divided into two main clades with distinct capsular loci: KL64 (74 %, DJ included, emerged in 1994 and disseminated worldwide, with carbapenemases varying among world regions) and KL10 (20 %, emerged in 2002, predominantly found in Asian countries, associated with carbapenemases NDM and OXA-48-like). Furthermore, subclades within ST147-KL64 differed at the yersiniabactin locus, OmpK35/K36 mutations, plasmid replicons and prophages. The absence of IncF plasmids in some subclades was associated with a possible activity of a CRISPR-Cas system. *K. pneumoniae* CG147 comprises pandrug-resistant or extensively resistant isolates, and carries multiple and diverse resistance genes and mobile genetic elements, including chromosomal *bla*_NDM-5_. Its emergence is being driven by the spread of several phylogenetic clades marked by their own genomic features and specific temporo–spatial dynamics. These findings highlight the need for precision surveillance strategies to limit the spread of particularly concerning CG147 subsets.

## Data Summary

Sequence read files and the complete genome assembly of isolate DJ have been deposited in the European Nucleotide Archive under the BioProject number PRJEB41234.

**Impact Statement**

*
Klebsiella pneumoniae* is currently emerging as one of the major antimicrobial-resistant bacterial pathogens that threaten public health. Multidrug-resistant *K. pneumoniae* have rapidly spread worldwide, a phenomenon mainly driven by the successful dissemination of a few particular high-risk sublineages, such as clonal group (CG) 258, CG307 and CG147. Whereas the evolutionary history of emergence of CG258 and CG307 have been studied in details, that is not the case for CG147. In this study, we characterized a CG147 pandrug-resistant strain isolated in 2016 in India and addressed the lack of knowledge on CG147 population emergence, providing unique insights into the genomic evolution and antimicrobial-resistance gene dynamics of this CG. The results highlight the power of population genomics in defining the most concerning subgroups (phylogenetic clades) within emerging high-risk *K. pneumoniae* clones, and provide a priority focus for surveillance and control strategies of particularly concerning clades. Moreover, the possible link between the absence of IncF plasmids in recent CG147 subclades and the activation of the CRISPR defence system, points out the need for more research on the mechanistic drivers of the flux of genetic elements in the bacterial lineages of public-health concern.

## Introduction

The increasing number of antimicrobial-resistant *Klebsiella pneumoniae* infections, especially by extended-spectrum β-lactamase (ESBL)- and carbapenemase-producing *K *. *pneumoniae *, led to the declaration of *K *. *pneumoniae* as an ‘urgent threat’ and ‘priority pathogen’ by public health agencies [1, 2]. Molecular analyses of *K *. *pneumoniae* isolates has evidenced that the rapid emergence of multidrug-resistant (MDR) *K *. *pneumoniae* is largely driven by the geographical spread of successful clonal groups (CGs; e.g. CG15, CG101, CG147, CG258, CG307) [3], some of them carrying epidemic resistance plasmids [4]. In order to treat MDR *K *. *pneumoniae* infections, last-resort drugs such as polymyxins (especially colistin) and tigecycline are used [3, 5]. Consequently, resistance is also observed to these last-resort drugs, especially to colistin, and may culminate in the emergence and spread of pandrug-resistant strains [5, 6]. Pandrug-resistant *K *. *pneumoniae* strains leave few or no therapeutic options and are associated with high mortality rates [7–11].

The 7-gene multilocus sequence typing (MLST) sequence type (ST) ST147 has been recognized as a globally distributed antimicrobial-resistance clone [12], and is closely related to ST273 and ST392, which themselves comprise MDR isolates. Based on genomic classifications, these three STs are grouped into CG147 [13, 14]. The earliest studies of CG147 date from 2008 to 2010 in Hungary, and correspond to ciprofloxacin-resistant CTX-M-15-producing ST147 isolates, which had been disseminating in the country since 2005 [15, 16]. Between 2010 and 2014, CG147 (mainly ST147) was described worldwide in association with several carbapenemases [17]. Most reported CG147 isolates are from clinical samples, although some were found in companion animals, chimpanzees, poultry and poultry environments, and river waters [18–24]. The above studies were locally restricted and, so far, no study of the global spread and genome dynamics of this clone has been performed.

Here, we report a pandrug-resistant clinical isolate from India belonging to ST147, and investigate the genomic evolution and antimicrobial-resistance gene dynamics in the global CG147 population. We also analyse the phylogenetic context of virulence-associated genomic features, CRISPR-Cas loci and mobile genetic elements (MGEs) in this successful *K *. *pneumoniae* sublineage.

## Methods

### Isolation and phenotypic and genomic characterization of strain DJ

Isolate DJ was recovered from the urine of a 45-year-old female patient diagnosed with a urinary tract infection in Vadodara (Gujarat, India) in October 2016. The isolate was confirmed to be *K *. *pneumoniae* by biochemical tests and 16S rRNA sequencing. Antibiotic-susceptibility tests performed using a semi-automated commercial system (Vitek; bioMérieux) revealed resistance to all antibiotics tested. Confirmatory antimicrobial-susceptibility tests were carried out. For colistin and tigecycline we used broth dilution, whereas for fosfomycinwe performed the agar dilution method (agar supplemented with 25 mg l^−1^ glucose 6-phosphate sodium salt). The disc diffusion method was used for the remaining antimicrobial classes (penicillins, cephalosporins, carbapenems, monobactams, fluoroquinolones, aminoglycosides, macrolides, tetracyclines, phenicols and inhibitors of the folic acid pathway). Results were interpreted using both the Clinical and Laboratory Standards Institute [25] and the European Committee on Antimicrobial Susceptibility Testing (2018) (http://www.eucast.org/) guidelines.

DNA extraction was performed using an XpressDNA bacteria kit (MagGenome Technologies). Whole-genome sequencing data were generated using: (i) an Illumina NextSeq-500 platform with a 2×150 nt paired-end protocol (Nextera XT library; Illumina); and (ii) long-read Oxford Nanopore sequencing using a MinION device integrated with a FLO-MIN-106 flow cell and libraries prepared using a 1D ligation sequencing kit (SQK-LSK109) following the protocol for 1D genomic DNA long reads without BluePippin (Oxford Nanopore Technologies). *De novo* assemblies of the reads were obtained using SPAdes v3.12.0 [26] for Illumina data and using Unicycler v0.4.4 [27] for hybrid assembly. Assembled sequences were annotated using Prokka v1.12 [28]. Reads and assembly data were deposited at the European Nucleotide Archive database (under BioProject accession no. PRJEB41234).

### Global dataset of publicly available genomic sequences

All publicly available CG147 *K. pneumoniae* genomes from the National Center for Biotechnology Information (NCBI) RefSeq repository of genome assemblies (May 2019) were downloaded. From the 245 CG147 genomes available, duplicated (*n*=5) and poor-quality genomes (*n*=6; genome size and G+C content not matching with *K. pneumoniae* and/or more than >1000 contigs), and those without an attached isolation year (*n*=17), were excluded. The final dataset comprised 218 genomes, including strain DJ. Sample information, accession numbers and biological characteristics of the genomes are given in Table S1 (available with the online version of this article).

### Phylogenetic analyses

For phylogenetic analyses, a core-genome alignment based on the concatenation of 4529 core genes was obtained using Roary v3.12 [29] using a blastp identity cut-off of 90 % and core genes defined as those being present in more than 90 % of the genomes. Recombination events were removed from the core-genome alignment using Gubbins v2.2.0 [30]. The final recombination-free alignment comprised 8450 single-nucleotide variants (SNVs) and was used to reconstruct a maximum-likelihood phylogenetic tree using iq-tree v1.6.11 (model GTR+F+ASC+G4). The tree was rooted with a *K. pneumoniae* ST258 NJST258_2 (accession no. GCF_000597905.1) and a *K. pneumoniae* ST37 INF042 (accession no. GCF_002752995.1) (Fig. S1).

To evaluate the strength of the temporal signal of our molecular phylogeny, we first conducted a linear regression analysis of the root-to-tip genetic distances as a function of the sample collection year, using TempEst v1.5.3 (http://tree.bio.ed.ac.uk/software/tempest/) (Fig. S2). The final recombination-free alignment was then subjected to Bayesian phylogenetic analysis using beast v2.6.1 (run with a Markov chain Monte Carlo length of 1×10^9^, sampling every 5×10^3^ steps) [31]. We used model parameters that had the best fit: GTR substitution model, lognormal relaxed clock and constant population size. Parameter estimates were computed using Tracer v1.7.1, and a maximum clade credibility tree was obtained with TreeAnnotator v2.6.0. and visualized in FigTree v1.4.4.

### MLST and genomic analyses of resistance, virulence and other genetic elements

MLST (7 genes) was performed using the Institut Pasteur *Klebsiella* MLST [32] database (https://bigsdb.pasteur.fr/klebsiella/). Kleborate [33] and BIGSdb analytical tools (https://bigsdb.pasteur.fr/klebsiella/) [34] were used to define the presence of antimicrobial-resistance, heavy-metal-tolerance and virulence genes, and to characterize the capsular and liposaccharide O-antigen loci. Geneious Prime 2019.1.1 software (https://www.geneious.com) was used for further manual curation of antibiotic-resistance genes, and ISFinder (https://isfinder.biotoul.fr) was used to look for the insertion sequences in the resistance genes or in their genetic context. Plasmid replicons were detected using PlasmidFinder (https://cge.cbs.dtu.dk/services/PlasmidFinder/) [35], whereas prophages, integrative and conjugative elements (ICEs) and CRISPR-Cas systems were identified using phaster (https://phaster.ca) [36], ICEfinder (https://bioinfo-mml.sjtu.edu.cn/ICEfinder/index.php) and CRISPRCasFinder (https://crisprcas.i2bc.paris-saclay.fr/CrisprCasFinder/Index) [37], respectively. To depict co-resistance genotypes and plasmid networks, we constructed a correlation matrix for binary variables (1, presence; 0, absence) using the ‘corr.test’ function (Pearson method, which for a pair of binary variables compares to the Phi coefficient) from the ‘corrplot’ R package. Significant correlations were visualized with the corrplot function from the same package. Statistical analyses to check the association of the different categorical variables within the phylogeny groups were calculated using the *χ* ^2^ test (*P* values of <0.05 were considered statistically significant).

## Results

### Phenotypic and genomic features of pandrug-resistant strain DJ

Strain DJ was resistant to all tested antimicrobial agents, including last-resort antimicrobials such as carbapenems, colistin, tigecycline and fosfomycin (Table 1), and is therefore pandrug resistant [6]. To define its genetic mechanisms of resistance, a hybrid complete genome assembly was produced (Fig. S3a). The 5.7 Mb sequence was 56.9 mol% G+C rich and made up of one chromosome and six circularized plasmids [123 kb IncFII(pKPX1); 57 kb IncR; 5.6 kb ColRNAI; 4.7 kb ColRNAI; 2.0 kb ColpVC; and 1.5 kb ColMG828]. In addition, there were two non-circularized contigs: a 84 kb IncFII plasmid and a 57 kb contig corresponding to a N15-*like* phage-plasmid (P-P) encoding a protelomerase (*telN*) responsible for the maintenance of its linear genome [38]. phaster identified four other prophages within the chromosome. Phylogenetic analysis of strain DJ showed it belonged to *K. pneumoniae sensu stricto* (i.e*.* phylogroup Kp1) and to the ST147-KL64 lineage previously described as endemic in India [12].

**Table 1.** Antimicrobial susceptibility of *K. pneumoniae* strain DJ, and genes potentially conferring resistance

Class and antimicrobial agent	Diameter (mm) or MIC (μg ml^−1^)	Interpretation*	Associated resistance genes (copy no.)
**β-Lactams**			*bla*_NDM-5_ (2), *bla*_OXA-181_ (2), *bla*_CTX-M-15_ (3), *bla*_TEM-1_ (2), *bla*_SHV-11_ (1), disrupted *ompK35,* mutation in *ompK36*
Ampicillin	*d*=9	R	
Piperacillin	*d*=13	R	
Amoxicillin–clavulanic acid	*d*=11	R	
Ticarcillin–clavulanic acid	*d*=14	R	
Piperacillin–tazobactam	*d*=14	R	
Cefuroxime	*d*=11	R	
Cefotaxime	*d*=12	R	
Ceftazidime	*d*=15	R	
Cefepime	*d*=15	R	
Aztreonam	*d*=14	R	
Ertapenem	*d*=12	R	
Imipenem	*d*=14	R	
Meropenem	*d*=15	R	
**Aminoglycosides**			*rmtB* (1)*, rmtF* (2), *aac(6’)-Ib* (2), *aadA2* (3), *strAB*(2)
Amikacin	*d*=10	R	
Gentamicin	*d*=10	R	
Tobramycin	*d*=9	R	
**Quinolones and Fluoroquinolones**			*gyrA* and *parC* mutations
Nalidixic acid	*d*=11	R	
Ofloxacin	*d*=11	R	
**Folate pathway inhibitors**			*sul1* (3)*, sul2* (1)*, dfrA12* (3)
Trimethoprim–sulfamethoxazole	*d*=19	R	
Trimethoprim	*d*=8	R	
**Polymyxins**			Disrupted *mgrB*
Colistin	MIC=4	R	
**Tetracyclines**			*ramR* mutation†
Tigecycline	MIC=16	R	
**Phenicols**			*catA2* (1), *catB* (2)
Chloramphenicol	*d*=12	R	
**Other**			
Fosfomycin	MIC=128	R	*fosA*
Rifampicin	NI	–	*arr-2* (2)
Macrolides	NI	–	*mphA* (1), *ermB* (1)

NI, Not included in the antimicrobial-susceptibility testing panel; R, resistant.

*Interpretations were based on Clinical and Laboratory Standards Institute (CLSI) and European Committee on Antimicrobial Susceptibility Testing (EUCAST) guidelines.

†Nucleotide mutation resulting in a premature stop codon.

**Fig. 1.**
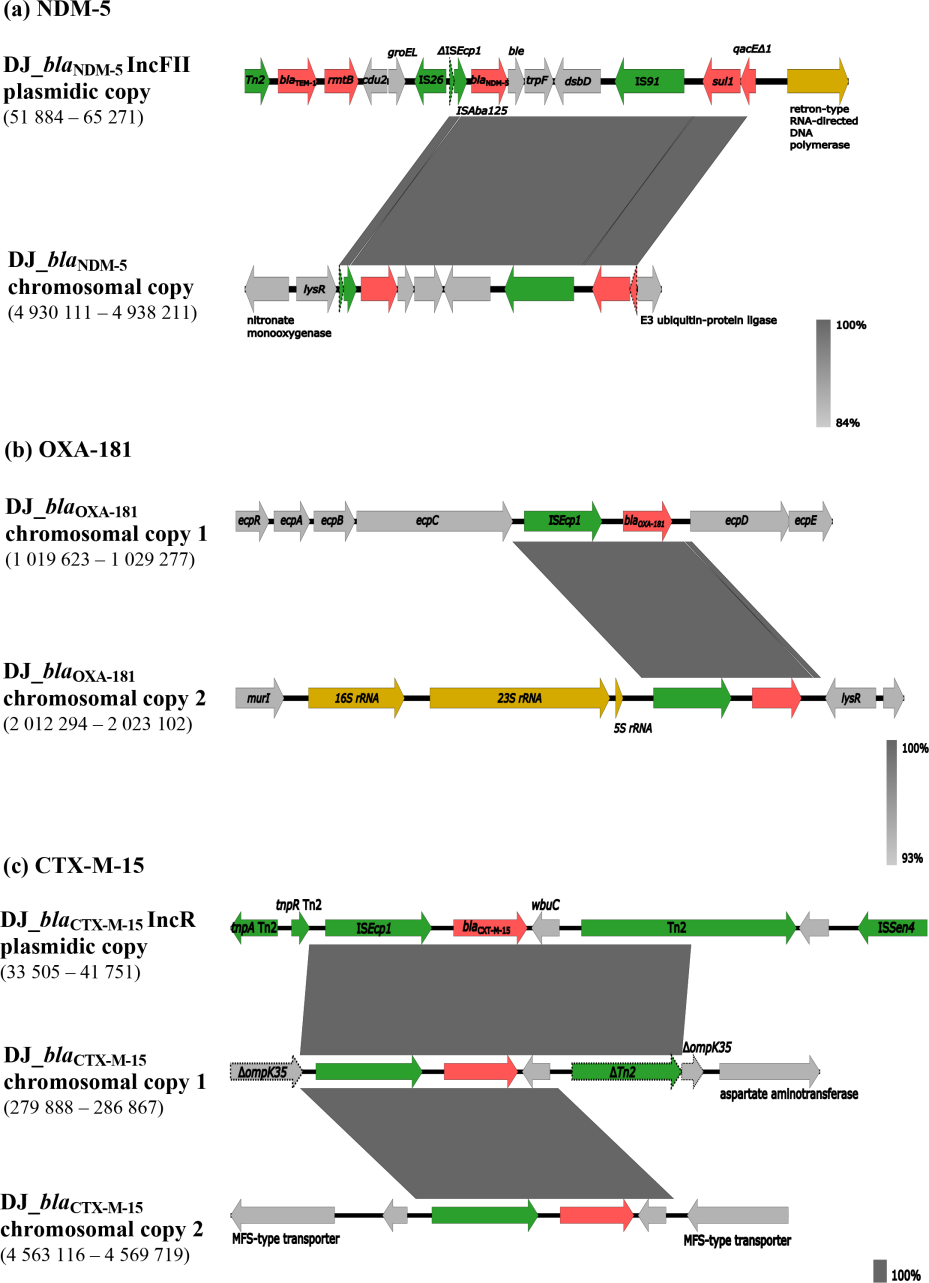
Genetic context of the different copies of *bla*_NDM-5_ (a), *bla*_OXA-181_ (b) and *bla*_CTX-M-15_ (c) genes identified in *K. pneumoniae* strain DJ. Within each panel, the chromosomal and/or plasmid-encoded copies of *bla*_NDM-5_, *bla*_OXA-181_ and *bla*_CTX-M-15_ detected in DJ are compared. Predicted ORFs are represented on each line by coloured arrows, with arrowheads indicating the direction of transcription: antimicrobial-resistance genes (red); MGEs or gene-mobilization-related genes (green); RNA-binding proteins (dark yellow); other functions (light grey). Disrupted genes are outlined with dotted lines. Dark grey blocks connecting the distinct gene regions represent homology levels, as indicated in the gradient key. The nucleotide positions of the represented regions are indicated on the left-hand side below the copy descriptors. Figures were created using Easyfig (https://mjsull.github.io/Easyfig/).

The long-read sequencing followed by hybrid assembly of strain DJ allowed the identification of multiple copies of unique antibiotic-resistance genes in the different genomic elements depicted (chromosome and plasmids). Strain DJ harboured four carbapenemase genes, corresponding to two copies of each of *bla*_NDM-5_ and *bla*_OXA-181_. Additionally, three copies of *bla*_CTX-M-15_ were detected (Table 1). The above genes were localized as follows (Figs 1 and S3a–e): (i) *bla*_NDM-5_ – one copy in the chromosome, and the second copy in the 84 kb IncFII plasmid; (ii) *bla*_OXA-181_ – two copies in the chromosome; and (iii) *bla*_CTX-M-15_ – two copies in the chromosome and one copy in the 57 kb IncR plasmid. In addition, a 61 kb region from the IncFII plasmid, comprising several antimicrobial-resistance genes included in a class one integron and the replication machinery of the plasmid, was duplicated in the chromosome (Fig. S3a, c).

Additional molecular determinants of antimicrobial resistance were observed in strain DJ. Most notably, the gene *mgrB* was disrupted by an IS*5* transposase (1057 bp), consistent with the observed colistin-resistance phenotype (MIC=4 µg ml^−1^). This strain also carried *rmtF, rmtB, strA, strB* and *aadA2* genes, associated with resistance to aminoglycosides including amikacin (Table 1). Quinolone resistance determining region (QRDR) mutations were observed, leading to GyrA-S83I and ParC-S80I amino acid alterations. Last, there was a premature stop codon caused by an A580T substitution in RamR, a negative regulator of RamA, itself a transcriptional activator of the *acrAB* genes. Higher production of AcrAB increases the efflux of tigecycline [39], consistent with the resistance phenotype observed for this agent (MIC=16 µg ml^−1^).

Regarding virulence genes, strain DJ harboured a complete yersiniabactin gene cluster (*ybt*10) located on an ICE*Kp*4 MGE (Fig. S3a). Type 1 (*fimAICDFGH*) and type 3 (*mrkABCD*) fimbriae gene clusters were also observed, but none of the *rmpACD*, aerobactin and salmochelin cluster genes, typically associated with hypervirulence, were present.

### Time-scaled phylogenetic structure of the global population of sublineage CG147

**Fig. 2.**
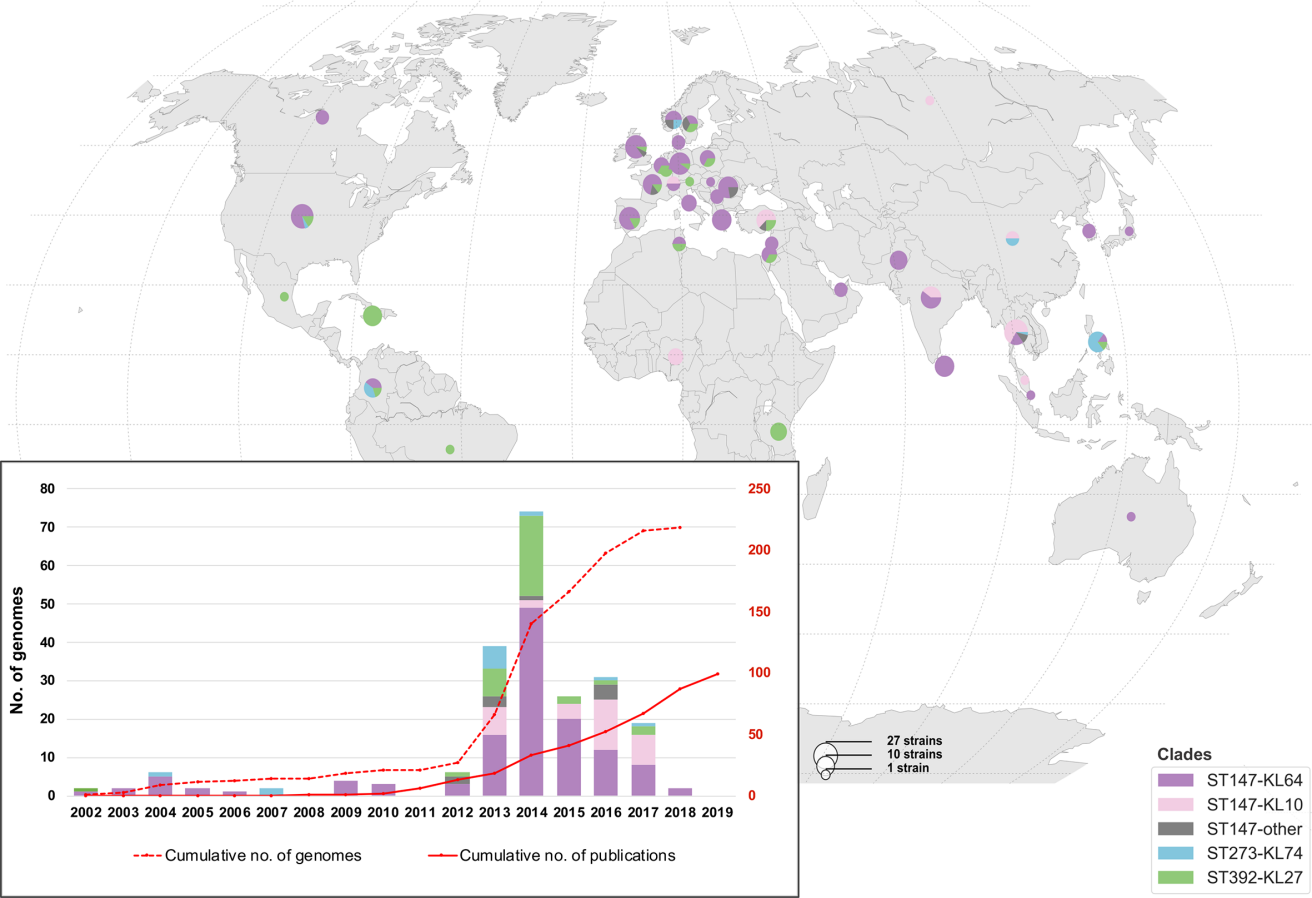
Geographical (main image) and temporal (inset) distribution of genomes included in this study. The pie charts represent the frequency of each CG147 clade in each country (see size and colour keys). Inset: bars represent the number of isolates per year for which genome assemblies were available (NCBI RefSeq) as of May 2019, coloured by clade. Red lines represent: (solid line) the number of PubMed-indexed records as of March 2020 (identified using the search criteria ‘* Klebsiella pneumoniae *’ and ‘ST147’ or ‘ST392’ or ‘ST273’, resulting in a total of 99 distinct entries); and (dotted line) the cumulative number of genomes. The scale on the left-hand side *y*-axis refers to total number, whereas the one on the right-hand side refers to cumulative numbers.

We investigated the evolutionary origins of strain DJ within the global diversity of CG147 using 217 publicly available genomes of isolates collected between 2002 and 2018. CG147 genomes were mainly isolated from human samples (90 %, 196/218) and hospital environments (6 %, 12/218), mostly in European (40 %), south-eastern Asian (17 %), southern Asian (11 %) and northern American (10 %) countries (Fig. 2).

**Fig. 3.**
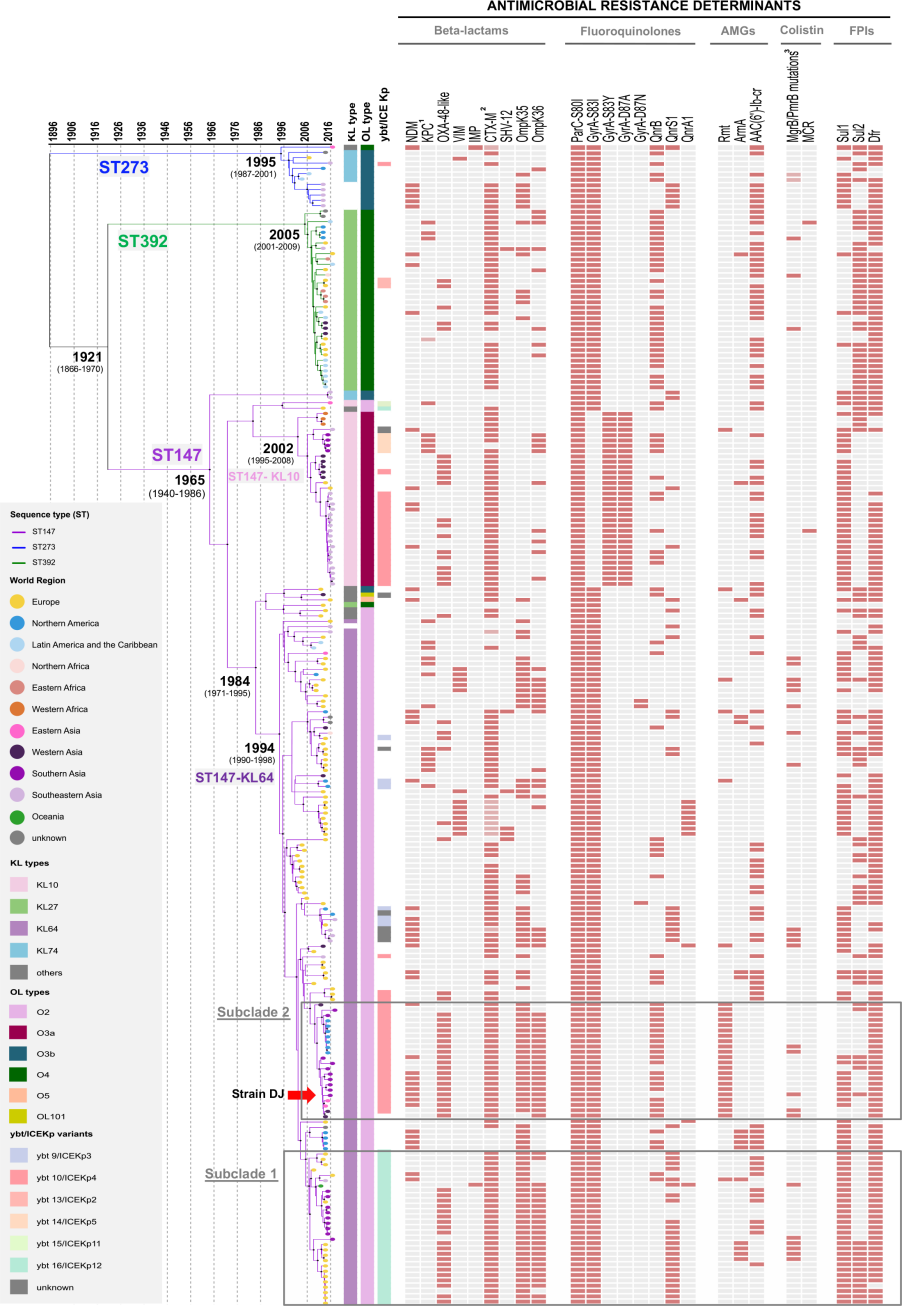
Time-scaled phylogeny of 218 CG147 genomes and their epidemiological and molecular characteristics. The phylogeny was obtained using the beast tool. The three main branches correspond to the three main STs. Tree tips are coloured by world region of isolation (see key). Black dots on main nodes indicate ≥95 % posterior probability. The grey boxes delineate subclades 1 and 2, as indicated. Capsular (KL) and O-antigen (O) locus types and the yersiniabactin-carrying ICE*Kp* elements are coloured according to their variants as shown in the key. Antimicrobial-resistance determinants are indicated by coloured rectangles when present. In the antimicrobial-resistance-determinant columns: 1, dark pink indicates *bla*_KPC-2_ and light pink indicates *bla*_KPC-3_; 2, dark pink indicates *bla*_CTX-M-15_ and light pink indicates other *bla*_CTX-M_ variants; 3, dark pink indicates *mgrB* mutations and light pink indicates *pmrB* mutations. AMGs, Aminoglycosides; FPIs, folate pathway inhibitors.

CG147 was deeply structured into three main branches (Fig. S1), each corresponding to a single MLST ST: ST147, ST273 and ST392 (the latter two are *tonB* variants of ST147). The number of genome-wide nucleotide substitutions was associated with isolation dates (root-to-tip regression analysis: *R* ^2^=0.1123; Fig. S2), enabling the inference of a time-scaled phylogeny (Fig. 3). The evolutionary rate within CG147 was estimated at 1.45×10^−6^ substitutions/site/year [95 % highest posterior density (HPD), 1.12x10^−6^ – 1.78×10^−6^], corresponding to 6.2 SNPs per genome per year. The last common ancestor of CG147 was estimated around year 1896, with a large uncertainty (95 % HPD: 1817–1962). The ST273 lineage was the first to diverge, whereas ST147 and ST392 shared a common ancestor, estimated around 1921 (95 % HPD, 1868–1970) (Fig. 3).

ST147 was the most represented (79 %, 172/218 genomes) and geographically widespread lineage (Fig. 2), and emerged around 1965 (95 % HPD, 1940–1986) (Fig. 3). The phylogenetic structure within ST147 revealed five main clades characterized by distinct capsular (KL type) and liposaccharide O antigen loci. Clades KL64-O2 (74 %, 128/172) and KL10-O3a (20%, 34/172) emerged in recent years: 1994 [95 % HPD, 1990–1998] and 2002 (95 % HPD, 1995–2008), respectively (Fig. 3). Whereas KL64-O2 genomes were predominantly from Europe (54 %), KL10-O3a was mainly sampled from Asia (85 %; Fig. 2).

The ST392 and ST273 branches emerged in 2005 (95 % HPD, 2001–2009) and 1995 (95 % HPD, 1987–2001), respectively (Fig. 3). ST392 (16 % of genomes, 34/218) is distributed globally and harbours a KL27 capsular gene cluster and a O4 antigen. In contrast, ST273 (6 %, 12/218) was predominantly found in Asia (64 %), and carries KL74 and O3b gene clusters (Figs 2 and 3). Of note, a group of closely related genomes from the Philippines (*n*=5) lacked a capsular gene cluster, with only *ugd* being detected. This gene had 100% identity with the *ugd* gene from the KL74 reference strain, suggesting a recent loss of the capsular gene cluster.

### Acquired antimicrobial-resistance genes and their evolutionary dynamics within CG147

All genomes presented QRDR alterations in GyrA and ParC. The topoisomerase ParC S80I alteration was fully conserved, whereas the GyrA gyrase subunit S83I amino acid change was observed in all genomes except for clade ST147-KL10, which had S83Y, caused by an ATC to TAC codon change. In addition, this clade had a D87A alteration (Fig. 3).

The number of acquired antimicrobial-resistance genes or mutations among CG147 genomes ranged from 2 to 23 (mean, 13; Table S1). Regarding β-lactam resistance, in addition to the conserved chromosomal *bl*a_SHV-11_ gene, a majority of CG147 isolates carried *bla*_CTX-M_ (*n*=181, 83 %), 94 % of which were the *bla*_CTX-M-15_ variant. In addition, 63 % (*n*=137) genomes harboured at least one carbapenemase gene, with 14 % (*n*=19) of these harbouring more than one copy of the same carbapenemase gene, and/or two or more carbapenemase genes from different families (Table S1). Of note, carbapenemase genes were significantly more frequent in ST147 (69 %) compared to ST392 (35 %) and ST273 (58 %; *P=*0.0005). The two main ST147 clades were similar in this respect (ST147-KL10, 74 %; ST147-KL64, 71 %; *P=*0.78). However, their carbapenemase genes were distinct across world regions: there was a predominance of *bla*_NDM_ in south-eastern Asia and northern America, whereas the combination of *bla*_NDM_ and *bla*_OXA-48-like_ was almost exclusively detected in south-eastern Asia, as observed in strain DJ. In contrast in Europe, *bla*_OXA-48-like,_ *bla*_KPC-2_ and *bla*_VIM-1/-27_ were the most frequent carbapenemases (Fig. S4, Table S1).

Different y*bt*/ICE*Kp* subtypes (associated with hypervirulence) and OmpK35/K36 mutations (associated with multidrug resistance) were observed within the ST147-KL64 clade (Fig. 3, Table S1). First, a group of 29 genomes (denominated subclade 1; mean number of SNPs among them, 57), which emerged around 2007, was characterized by the presence of *ybt*16/ICE*Kp*12 and an altered OmpK35 protein, due to a deletion of 2 nt resulting in a premature stop codon. Within this subclade itself, a subgroup of genomes (*n*=19/29) harboured *bla*_OXA-48-like_ genes and the OmpK36GD mutation observed previously [40]. Second, a group of 22 genomes emerged around 2009 (subclade 2; mean of 60 SNPs) was defined by the presence of *ybt*10/ICE*Kp*4 and an OmpK35 gene disrupted by IS*Ecp1-bla*_CTX-M-15_ (Figs 1 and 3, Table S1). As observed in subclade 1, all subclade 2 isolates carrying *bla*_OXA-181_ shared the same OmpK36TD mutation [40]. Subclades 1 and 2 also differed in plasmid replicon content: whereas the former were rich in IncR (90 %) and IncHIB/IncFIB(Mar) (41%), in contrast IncFII (pKPX1) (82 %), IncFII (59 %) and IncR (64 %) were frequent in the latter (Table S1). Differently, the remaining ST147-KL64 genomes (*n*=77) often carried IncFIB_K_ (65%), IncFII_K_ (49 %; a common pKPN-3-derived plasmid found in *K. pneumoniae* harbouring *pco* and *sil* clusters) [4, 41] and IncFIA(HI1) (38 %).

Strain DJ belonged to subclade 2 and was phylogenetically closely related (<28 SNPs) to five other isolates recovered between 2014 and 2015 in different Asian countries (Figs 3 and S3b) and described as extremely drug resistant or pandrug resistant [11, 42, 43], with identical plasmids being observed among them but no chromosomal integration of *bla*_NDM-5_ (Fig. S3a–e).

### Convergence of antimicrobial resistance and virulence

Whereas the yersiniabactin virulence factor gene cluster was rare amongst genomes of ST392 (6 %; 2/34) and ST273 (8 %; 1/12), it was observed in 53 % of ST147 genomes. There were two predominant variants (*ybt*16/ICE*Kp*12 and *ybt*10/ICE*Kp*4 associated with subclades 1 and 2, respectively), and four minority ones (Fig. 3, Table S1).

Two isolates with hypervirulence genotypes, defined by the presence of *rmpA* and/or *rmpA2* or aerobactin, were observed: KpvST147L (ST147-KL14, 2016, UK) and B-8658 (ST147-KL10, 2014, Russia). The plasmid from KpvST147L was fully sequenced previously (GenBank accession no. NZ_CM007852) and carries *rmpA*, *rmpA2* and aerobactin; it corresponds to a 343 kb IncFIB-IncHIB (pNDM-MAR) plasmid (Fig. S5) [44]. This plasmid was recently described in three ST147 *K. pneumoniae* isolates recovered in 2018–2019 in the UK and in other sublineages [45]. Here, we found that Russian strain B-8658 (ST147-KL10, 2014) also acquired the pNDM-MAR plasmid, but it lacked the *rmpA* gene (Fig. S5).

### Co-occurrence of antimicrobial-resistance and heavy-metal-tolerance genes and plasmids

Antimicrobial-resistance genes, mutations and plasmids co-occurred in a structured way (Fig. S6, Table S1). For example, Col(BS512), ColKp3, IncFIA (HI1), Cml, EreA/B and GyrA S83Y/D87A co-occurred frequently within the ST147-KL10 clade. Associations of (i) VIM, QnrA1, AadB and IncHI2, (ii) OXA-48-like and IncL(pOXA-48), or (iii) KPC and IncFIB(pQIL) were also observed, consistent with previous descriptions of genetic elements co-carrying these genes [46–48]. In addition, genes conferring tolerance to copper and silver were associated with IncFII_K_/IncFIB_K_ plasmids (*pco* and *sil* operons; *P*<0.00001), whereas genes for mercuric resistance were linked to IncR plasmids (*P*=0.0082). Similarly, a positive association between the tellurite cluster and IncHIB/IncFIB (MAR) was observed (*P*<0.0001) [4]. Last, a negative association between IncR and IncFII_K_/IncFIB_K_ was observed.

### CRISPR-Cas systems

We investigated whether resistance gene dynamics could be influenced by CRISPR-Cas systems in CG147. There were either one (83 %; 180/218) or two (17 %; 37/218) CRISPR-Cas systems amongst CG147 genomes. A conserved subtype I-E system was found in all genomes, located in the *iap–cysH* region, with a 60.6 mol% G+C content, and defined arbitrary as CRISPR1. Direct repeat sequences were highly conserved, but the number and sequences of spacers varied across CG147 genomes. CRISPR1 variant v0 (43 spacers) was present in 56 % of the genomes (122/218) and was used as reference to define CRISPR1 variants (Fig. S7, Table S2). Four of the CRISPR1 v0 spacers (spacers 1, 28, 29 and 42) matched sequences on MDR IncF plasmids disseminated among different *Enterobacteriaceae *, including *K. pneumoniae *. Spacer 1 matched a multicopy intergenic region, whereas spacers 28 and 29 matched a DUF3560-domain containing protein, and spacer 42 a hypothetical protein located upstream of SAM-methyltransferase. Although these spacers were highly conserved among CRISPR1 variants, they were also found at high frequency (in 28–53 % of the strains) as protospacers located in plasmid contigs. Of note, in 28 % (62/218) of CG147 genomes these plasmid protospacers were not detected, and the majority of these strains (65%, 40/62) belonged to subclades 1 and 2, which were also characterized by the absence of IncFII(K) and IncFIB(KpQIL) plasmid replicons. The association of the lack of IncF plasmids with IncF-targeting spacers in CRISPR1 suggests a possible activity of this CRISPR system in subclades 1 and 2. Finally, twelve of the forty-three spacers from CRISPR1 v0 targeted prophages; of these, spacers 6 and 25 were found as protospacers in some of the CG147 genomes (28 and 18 %, respectively).

Six other CRISPR-Cas systems, distinct from CRISPR1, were observed in 37 genomes. CRISPR2 to CRISPR4, of type IV-A3, were strongly associated with IncHIB and/or IncFIB (pNDM-MAR) plasmids (*P<*0.00001), as described elsewhere [49]. Two variants of CRISPR2 (v1, *n*=12, 17 spacers; and v3, *n*=5, 25 spacers) were prevalent (Fig. S7, Table S2). CRISPR2 to CRISPR4 systems shared identical direct repeat sequences, but showed a high diversity in the number and sequences of spacers. Their tendency to target IncFII_K_/IncFIB_K_ plasmids has led to a suggestion of a role in inter-plasmid competition [49, 50]. However, here, the presence of CRISPR2 to CRISPR4 systems was not uniformly associated with an absence of IncFII_K_/IncFIB_K_ plasmids.

### Prophage elements

CG147 genomes harboured zero to seven prophages (mean of four prophages per genome; considering only the intact ones). Some prophages were frequent, including (i) ST147-VIM1phi7.1-*like* (GenBank accession no. NC_049451; *Myoviridae* family), which was present in 90 % of the genomes (196/218); (ii) *Salmonella* phage 118970_sal3-*like* [GenBank accession no. NC_031940; *Myoviridae* family; 70 % (152/218)]; and (iii) *Enterobacteria* phage mEp237-*like* [GenBank accession no. NC_019704; *Siphoviridae* family; 56 % (121/218)] (Fig. S7).

The N15-*like* phage-plasmid (*Siphoviridae* family) we uncovered in the genomic assembly of strain DJ was present in 37 % of CG147 genomes (81/218). It was strongly associated (*P*<0.00001) with ST147-KL64 subclades 1 and 2, and present in 92 % of these genomes (Table S1, Fig. S7). These two subclades were also enriched in other prophages (mean number of prophages: 5) compared with the remaining CG147 genomes (mean number of prophages: 3).

## Discussion

This study was triggered by the discovery of a pandrug-resistance phenotype in strain DJ from India, which prompted us to analyse its genomic features and understand its dynamics in the context of a large genome dataset of CG147 from multiple world regions. The results revealed its deep phylogenetic structure and a capacity of CG147 members to acquire a wide range of antimicrobial-resistance or virulence elements, plasmids and prophages. Of these, *Klebsiella* capsular locus (KL) switches, which occurred repeatedly, represent prominent phylogenetic markers of CG147 clades. The evolutionary dynamics of these surface structures in CG147 echo those observed in other MDR CGs, such as ST258 or ST307 [13, 47, 51]. The multiple clonal expansions of sublineages with distinct KL or O types has clear implications for diagnostic, control or therapeutic strategies such as vaccination and phage therapy. Although its reliance on public genomic sequences may expose this study to a bias towards antibiotic-resistant CG147 isolates, as well as those involved in nosocomial outbreaks, this study provides new insights into the evolutionary history, epidemiology and population dynamics of this important emerging CG.

The genomic arsenal of antimicrobial-resistance features of subclade 2, to which strain DJ belongs, suggest extensively drug and pandrug resistance is not restricted to strain DJ, but rather is a shared characteristic in this particular subclade, already disseminated among Asian countries [11, 42, 43, 52–54]. Clonal spread of this subclade between different countries was reported [52]. This subclade should be closely monitored and may represent a pioneering situation ushering the worrying prospect of pan-resistance in other CG147 subclades.

In the case of strain DJ, we observed the integration of *bla*_NDM-5_ in the chromosome. Previous studies have reported one or multiple copies of *bla*_CTX-M-15_ and *bla*_OXA-181_ integrated into the chromosome of *K. pneumoniae* isolates [55, 56], including in the high-risk ST147 [52, 57]. However, the chromosomal integration of *bla*_NDM_ was, to our knowledge, only reported once in *K. pneumoniae *, in two NDM-1 producing ST14 clinical isolates from Thailand [58]. In that case, the chromosomal integration was mediated by IS*5* and the Tn*3* transposase. In strain DJ, the integration of *bla*_NDM-5_ may have been mediated by IS*Ecp*1/IS*26* (Fig. 1a). The genomic rearrangement due to the replicative transposition of IS*26* has previously been shown for IncFII-*bla*_NDM-5_ bearing plasmids [59]. The chromosomal incorporation of carbapenemase and ESBL genes is concerning, as it may stabilize these genes by promoting their vertical dissemination [52].

The convergence of MDR and hypervirulence genotypes is being increasingly observed in *K. pneumoniae* [3]. Here, this worrisome association was detected in two phylogenetically distinct CG147 isolates from 2014 (Russia) and 2016 (UK), which shared an MDR-Hv IncHIB/FIB plasmid [44]. The recent observation of this plasmid in ST101 and ST147 isolates from the UK [45], with no epidemiological link with the isolate from 2016, suggests its continuous circulation and further risk of horizontal spread.

The ST147-KL64 lineage is globally disseminated. The evolutionary rate we estimated (1.03×10^−6^ substitutions/site/year) is very similar to other MDR global sublineages, such as ST258 (1.03×10^−6^ substitutions/site/year) and ST307 (1.18×10^−6^ substitutions/site/year) [47, 51], and slightly slower than the one estimated for ST101 (2.85×10^−6^ substitutions/site/year) [60]. It is striking that the emergence of ST147-KL64 lineage occurred approximately at the same time as other MDR sublineages ST258 (year 1995), ST307 (1994) and ST101 (1989) [47, 51, 60]. In addition, the presence of GyrA and ParC QRDR alterations is a common characteristic to these MDR high-risk sublineages (CG258 and ST307 – GyrA-S83I and ParC-S80I; ST101 – GyrA-S83I, GyrA-D87G/N/A and ParC-S80I) [47, 51, 60]. This phenotypic and temporal conjunction points to common drivers and suggests a role of the usage of fluoroquinolones, introduced into clinical practice at the end of the 1980s, in the emergence of MDR *K. pneumoniae* sublineages. This is reminiscent of the scenarios of emergence of *Escherichia coli* ST131 and meticillin-resistant *Staphylococcus aureus* ST22 [61–63].

The drivers of genomic diversification of emerging *K. pneumoniae* sublineages may include a combination of ecological opportunities to acquire genetic elements, local selective pressure, and molecular mechanisms that enable or restrict genetic flux. Of these, CRISPR-Cas systems may play a role [64–67]. In CG258, an association was suggested between the absence of these systems and the ability to acquire IncF plasmids [such as *bla*_KPC_-IncF(pKpQIL-like plasmids)] [68–70]. Here, we found a conserved type I-E CRISPR-Cas system (CRISPR1) within CG147, consistent with early reports [9], with four spacers matching IncF plasmid sequences. The distribution of CRISPR1 in the broader *K. pneumoniae* species shows a unique association with CG147, with only two exceptions (ST2746 and ST3700; based on 1001 *K. pneumoniae* genomes representing unique STs; selected from a dataset of 4222 genomes from the NCBI, November 2018; data not shown). Type IV CRISPR-Cas systems primarily target plasmids [50]. However, in the majority of CG147 strains, corresponding protospacers were found in plasmid sequences, advocating that the immunity provided by this CRISPR1 system might not be fully functional. In contrast, ST147-KL64 subclades 1 and 2 were largely devoid of these protospacers, and did not carry IncFIA, IncFII_K_, IncFIB_K_ and IncFIB (pQil) plasmids (Fig. S7). This observation may suggest a possible activity of the CRIPSR1 system in these recently emerged subclades. Future experimental studies are needed to explore this hypothesis. Of note, it was also among these subclades that the N15-*like* phage-plasmid was prevalent. Among bacterial phyla, this phage-plasmid family was found almost exclusively in *K. pneumoniae* [38], and we found that 58.9 % of these belonged to CG147. A possible biological role of phage-plasmids has yet to be established.

### Conclusions

The presence of pandrug-resistant and extremely drug resistant isolates in CG147, together with the high genetic plasticity and rapid emergence dynamics of this clone, represents a clear threat to public health. CG147 is globally disseminated but shows a strong phylogenetic structure, with different clades being associated with specific genomic features and geographical distributions. These observations underline how different variants of CG147 contribute to the major public-health threat posed by *K. pneumoniae *, and call for specific surveillance and directed control strategies of this clone and its particularly concerning clades [12].

A possible link between the absence of IncF plasmids and the activation of the CRISPR1 defence system is intriguing. This observation calls for more work on mechanistic drivers of the flux of genetic elements across MDR bacterial lineages. Precise phylogenetic mapping and understanding of the dynamics of antimicrobial-resistance features are needed to guide the development of control strategies, especially those that target specific subsets of strains within pathogenic bacterial species, such as CRISPR delivery or toxic conjugation systems [71, 72].

## Supplementary Data

Supplementary material 1

Supplementary material 2
