# Supplementary material for: Genomic evolution of the globally disseminated multidrug-resistant Klebsiella pneumoniae clonal group 147
Source: Microb Genom. 2022 Jan 12;8(1):000737. doi: 10.1099/mgen.0.000737 (PMC8914359; doi:10.1099/mgen.0.000737)
Supplement: Supplementary material 1 [file mgen-8-0737-s001.pdf]

Supplementary Data

Genomic evolution of the globally disseminated multidrug-resistant *Klebsiella pneumoniae* clonal group 147

Carla Rodrigues[1], Siddhi Desai[2], Virginie Passet[1], Devarshi Gajjar[2] and Sylvain Brisse[1*]

[1] Institut Pasteur, Biodiversity and Epidemiology of Bacterial Pathogens, Paris, France.

[2] Department of Microbiology and Biotechnology Centre, Faculty of Science, The Maharaja Sayajirao University of Baroda, Vadodara, Gujarat, India.

***Corresponding author:**

Sylvain Brisse

Institut Pasteur,

Biodiversity and Epidemiology of Bacterial Pathogens,

28 rue du Docteur Roux, F-75724 Paris, France.

E-mail: sylvain.brisse@pasteur.fr;

Phone: +33 1 45 68 83 34

Contents

Figure S1. Phylogenetic structure of CG147.

Figure S2. Temporal signal in genomic sequences.

Figure S3. Replicons of strain DJ.

Figure S4. Geographic origins of the genomes included in this study and their associated carbapenemase profile.

Figure S5. Sequence alignments of MDR-Hv plasmids identified in CG147 genomes.

Figure S6. Correlation between genotypes (Pearson method).

Figure S7. Time-scaled phylogeny of CG147 genomes and their heavy metal tolerance genes, plasmid replicons, CRIPSR systems, and prophages profile.

Table S2. CRISPR/Cas systems and variants identified within CG147 *K. pneumoniae* genomes.

Please see separate Excel file for Table S1.

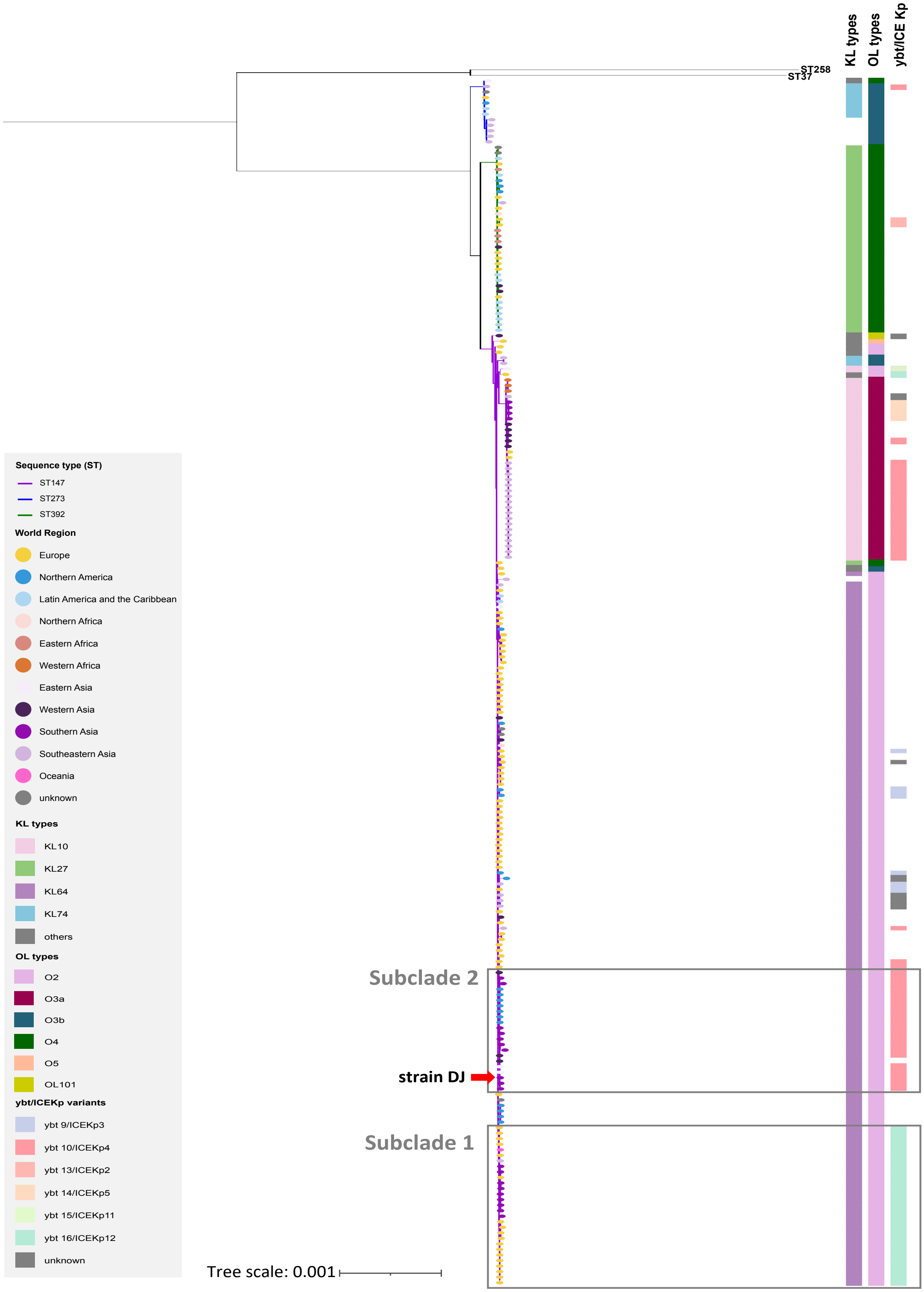

Figure S1. **Phylogenetic structure of CG147.**

The tree was obtained by maximum likelihood analysis (IQ-TREE) based on the final recombination-free alignment of concatenated nucleotide sequence alignments of 4,517 core genes. The tree was rooted with a Kp ST258 NJST258_2 (GCF_000597905.1) and a Kp ST37

INF042 (GCF_002752995.1). Branch lengths represent the number of nucleotide substitutions per site (scale, 0.001 substitution per site). The three main branches are colored by sequence type (ST) and branch tips are colored by world region of isolation (see key). Capsular (KL) and O-antigen (O) locus types and the yersiniabactin-carrying ICE*Kp* elements are colored according to their variants (see key). Subclades 1 and 2 are denoted by rectangle outlines.

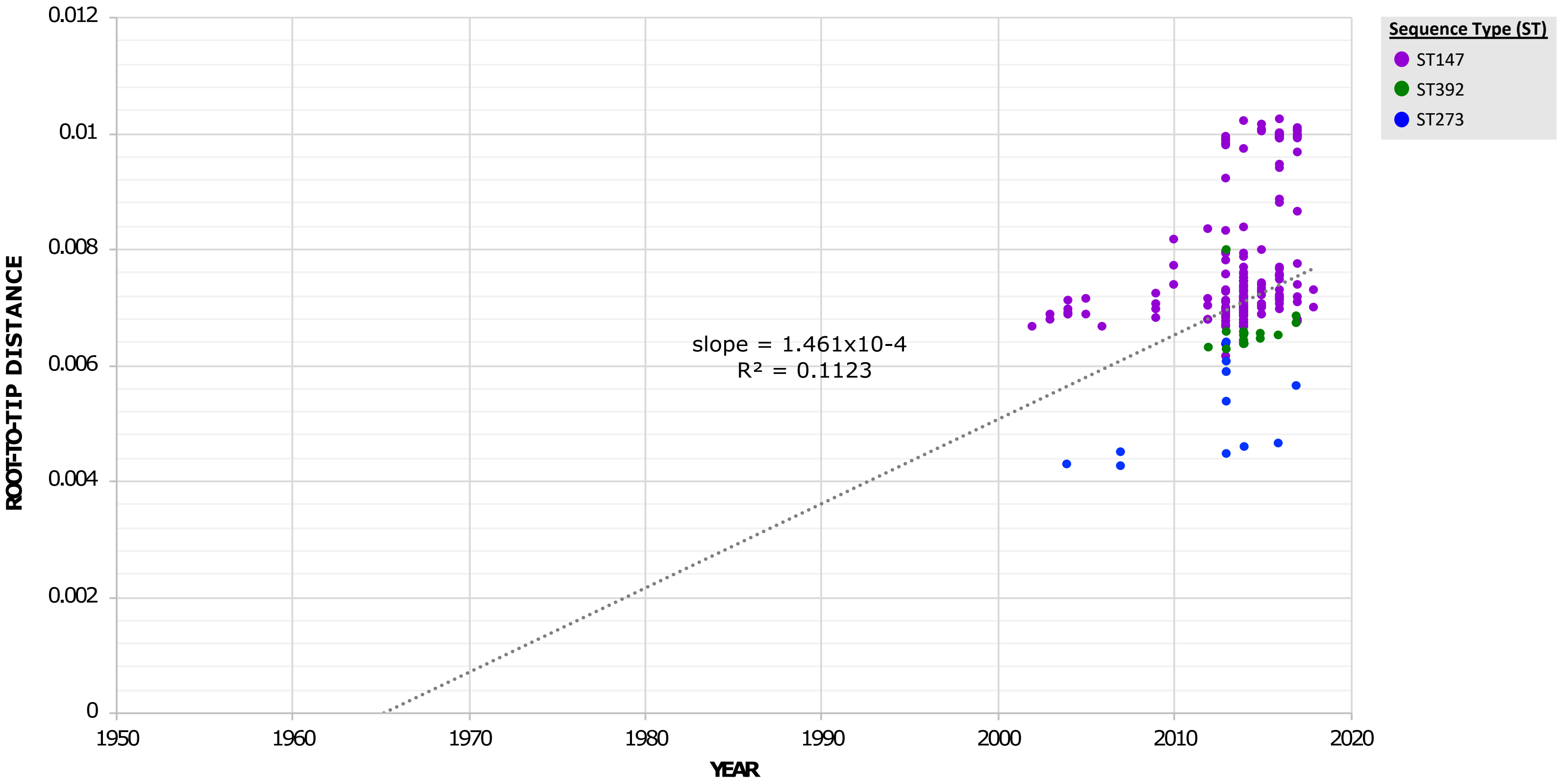

Figure S2. Temporal signal in genomic sequences.
The linear correlation between year of isolation and root-to-tip distance from the maximum likelihood phylogeny for CG147 genomes collection was calculated using TempEst. Genome dots are coloured by the Sequence Type (ST).

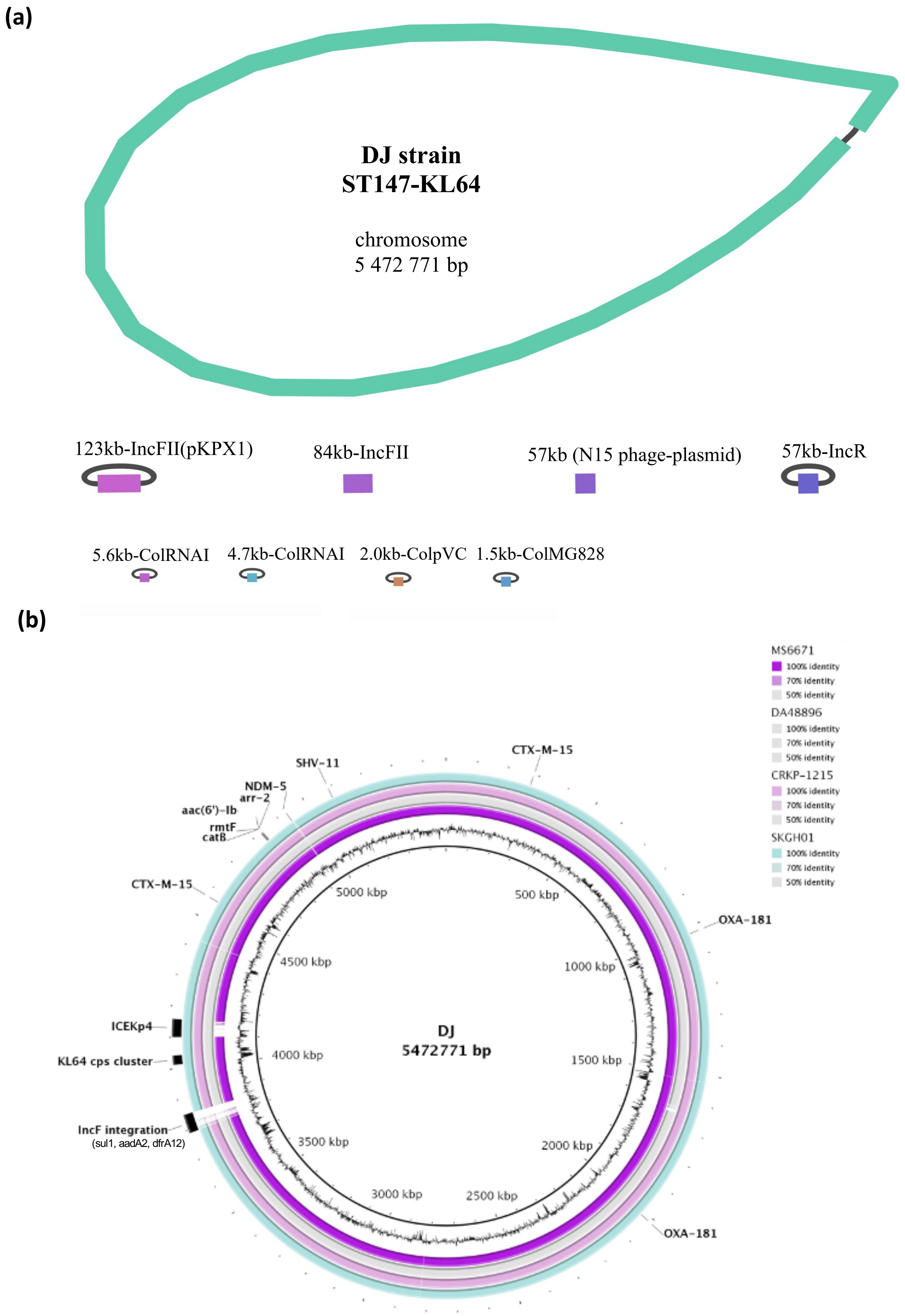

(a)
DJ strain
ST147-KL64
chromosome
5 472 771 bp
123kb-IncFII(pKPX1)
84kb-IncFII
57kb (N15 phage-plasmid)
57kb-IncR
5.6kb-ColRNAI
4.7kb-ColRNAI
2.0kb-ColpVC
1.5kb-ColMG828
(b)
MS6671
100% identity
70% identity
50% identity
DA48896
100% identity
70% identity
50% identity
CRKP-1215
100% identity
70% identity
50% identity
SKGH01
100% identity
70% identity
50% identity
SHV-11
CTX-M-15
NDM-5
arr-2
aac(6')-Ib
rmtF
catB
CTX-M-15
OXA-181
ICEKp4
KL64 cps cluster
IncF integration
(sul1, aadA2, dfrA12)
OXA-181
DJ
5472771 bp
5000 kbp
500 kbp
4500 kbp
1000 kbp
4000 kbp
1500 kbp
3500 kbp
2000 kbp
3000 kbp
2500 kbp

(c)

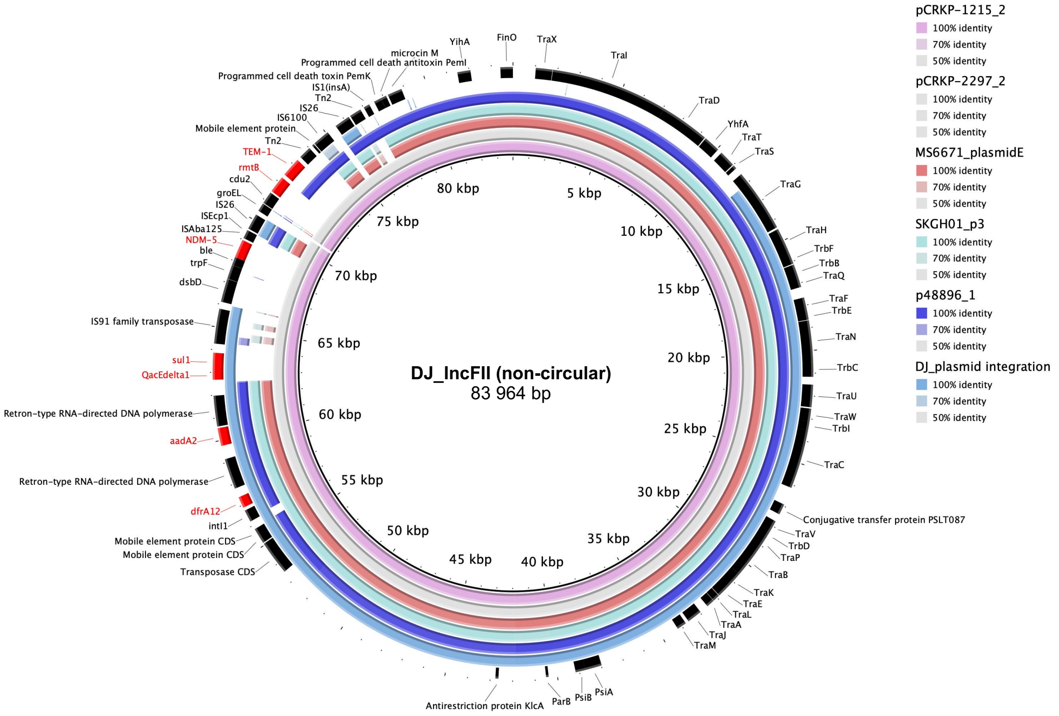

	Cov.	ID	Size (bp)	AMR genes
**pCRKP-2297_2** (IncFII)	99%	99.61%	96 185	***blaNDM−5, blaTEM−1B, dfrA12, aadA2, sul1, rmtB***, *mphA, ermB*
**pCRKP-1215_2** (IncFII)	99%	99.61%	96 185	***blaNDM−5, blaTEM−1B, dfrA12, aadA2, sul1, rmtB***, *mphA, ermB*
**SKGH01_p3** (IncFII)	85%	99.85%	84 941	*aac(6')-lb-cr; rmtF*, ***aadA2, dfrA12***
**MS6671_plasmidE** (IncFII)	85%	99.61%	84 940	*aac(6')-lb-cr; rmtF*, ***aadA2, dfrA12***
**p48896_1** (IncFII/IncR)	88%	99.61%	131 243	***blaCTX-M-15, blaTEM−1B, catA2, aadA2***, **dfrA12, strAB (2x), sul2**

(d)

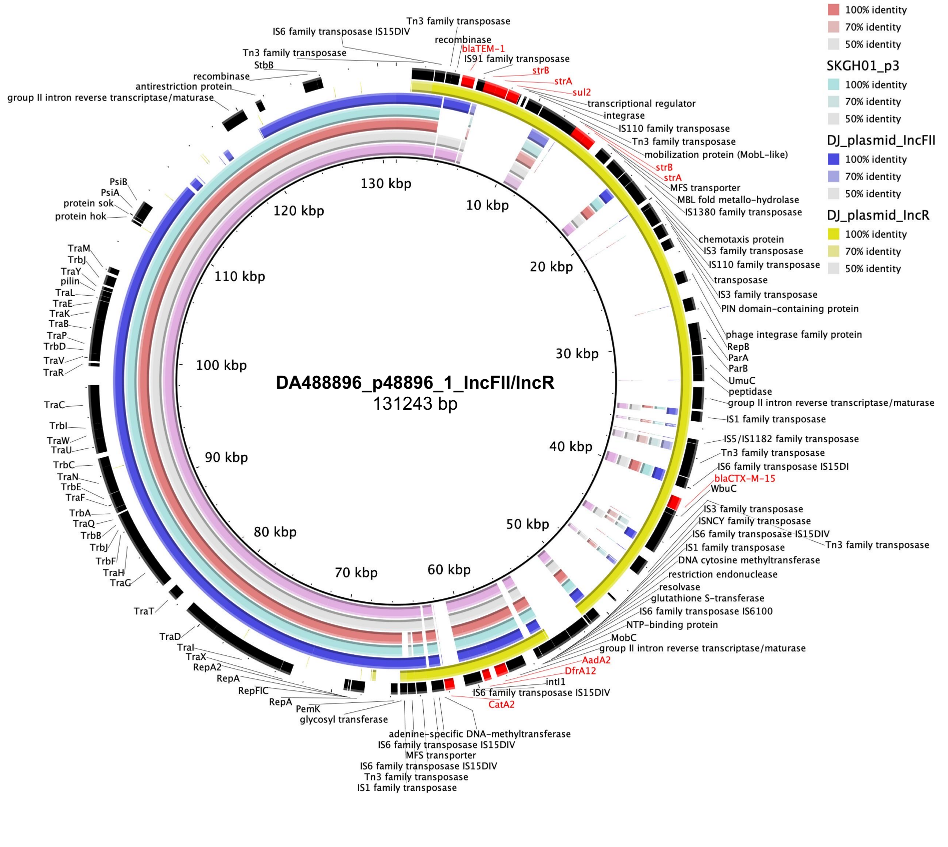

(e)

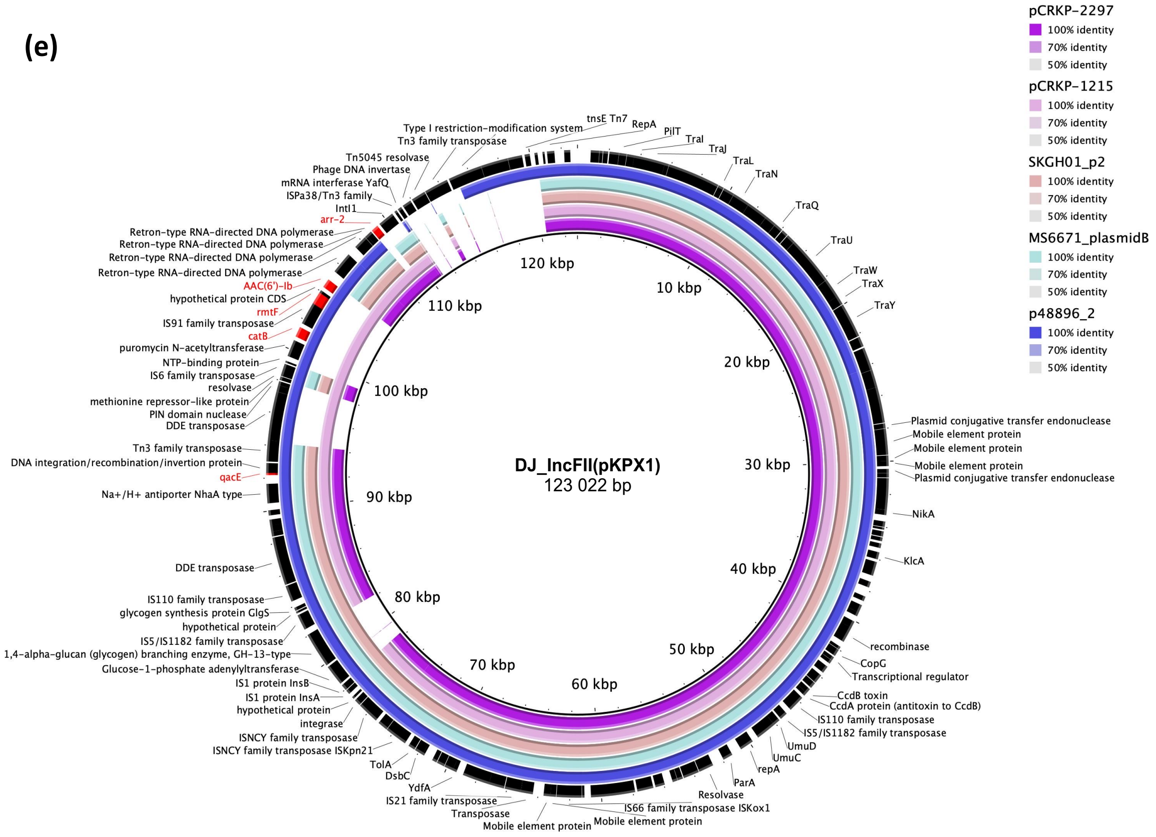

	Cov.	ID	Size (bp)	AMR genes
**pCRKP-2297_1**	81%	99.98%	112 150	***arr-2***, *qnrB1*
**pCRKP-1215_1**	89%	99.98%	130 922	***arr-2***, *qnrB1*, ***rmtF***, *aadA2*, ***aacA4,*** *dfrA12*, *catA2*
**SKGH01_p2**	84%	99.78%	140 874	*qnrB1*, *dfrA14*
**MS6671_plasmidB**	84%	99.96%	118 323	*qnrB1*, *dfrA14*
**p48896_2**	95%	99.97%	114 815	***rmtF, aacA4***

Figure S3. **Replicons of strain DJ.**

(**a**) Visual representation of the hybrid Unicycler assembly graph, obtained using Bandage (Wick et al., 2015). Nine replicons were obtained, including the circularized chromosome, 6 circularized plasmids, 1 linear phage-plasmid and 1 non-circularized plasmid contig. The size of replicons is shown.

(**b**) Sequence alignments of the chromosomes of strain DJ and the four closest genomes belonging to subclade 2 (<28 SNPs): MS6671 (GenBank Accession Number: GCA_001455995), DA48896 (GCA_003006175.1); CRKP-1215 (GCA_002786755; =CRKP-2297) and SKGH01 (GCA_001644765.1). Strain DJ was used as reference. The outermost circle is an annotation of the reference strain showing the location of antimicrobial drug resistance genes, ytb/ICEKp, KL64 capsular locus and the IncFII chromosomal integration region. The figure was created using BRIG (Alikhan et al., 2011).

(**c**) Sequence alignments of the non-circular IncFII replicon of strain DJ with the closest plasmids of subclade 2 strains: pCRKP-2297_2 (CP024836.1); pCRKP-1215_2 (CP024840.1), MS6671_plasmidE (LN824138.1), SKGH01_p3 (CP015503.1), p48896_1 (CP024430.1) and the region integrated in the chromosome of strain DJ. The IncFII non-circularized replicon was used as reference. The outermost circle is an annotation of the reference strain showing the location of antimicrobial drug resistance genes in red. In the table the coverage, identity, size and antimicrobial resistance genes of the plasmids that were used for the comparison. In bold the resistance genes also present in DJ replicon.
(**d**) Sequence alignments of the p48896_1 (CP024430.1) plasmid with the plasmids of the closest strains: pCRKP-2297_2 (CP024836.1); pCRKP-1215_2 (CP024840.1), MS6671_plasmidE (LN824138.1), SKGH01_p3 (CP015503.1), DJ-IncFII non-circularized replicon and DJ-IncR plasmid. p48896_1 was used as reference plasmid. The outermost circle is an annotation of the reference strain showing the location of antimicrobial drug resistance genes in red.
(**e**) Sequence alignments of the 123 kb IncFII(pKPX1) plasmid of strain DJ with the closest plasmids of subclade 2 strains: pCRKP-2297_1 (CP024835.1), pCRKP-1215_1 (CP024839.1), MS6671_plasmidB (LN824135.1), SKGH01_p2 (CP015502.1), p48896_2 (CP024431.1). The IncFII(pKPX1) plasmid of strain DJ was used as reference. The outermost circle is an annotation of the reference strain showing the location of antimicrobial drug resistance genes in red. In the table the coverage, identity, size and antimicrobial resistance genes of the plasmids that were used for the comparison. In bold the resistance genes also present in DJ replicon.

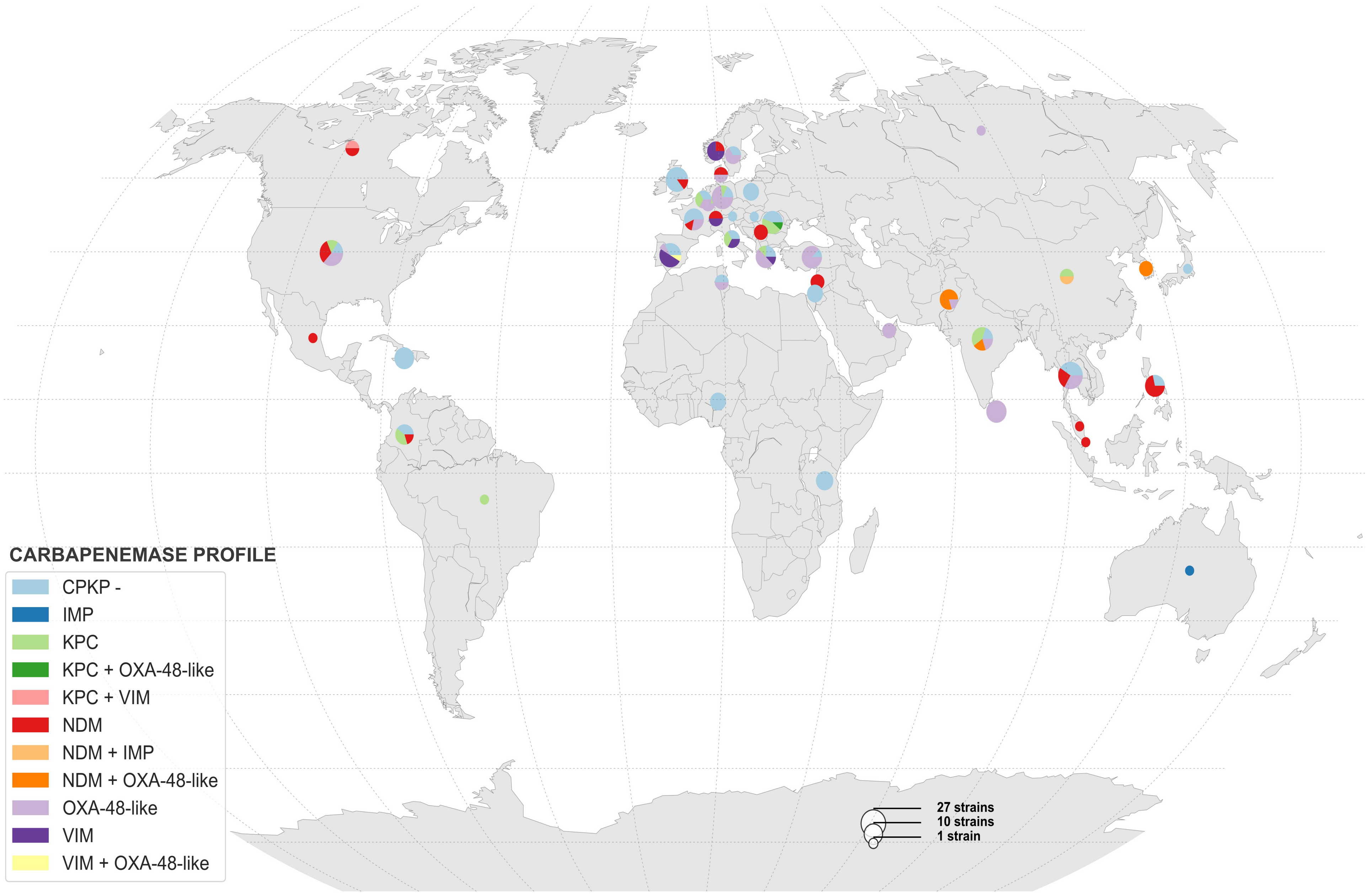

Figure S4. **Geographic origins of the genomes included in this study and their associated carbapenemase profile.** The pie charts represent the frequency of each carbapenemase profile in each country (see key). CPKP-: no carbapenemase.

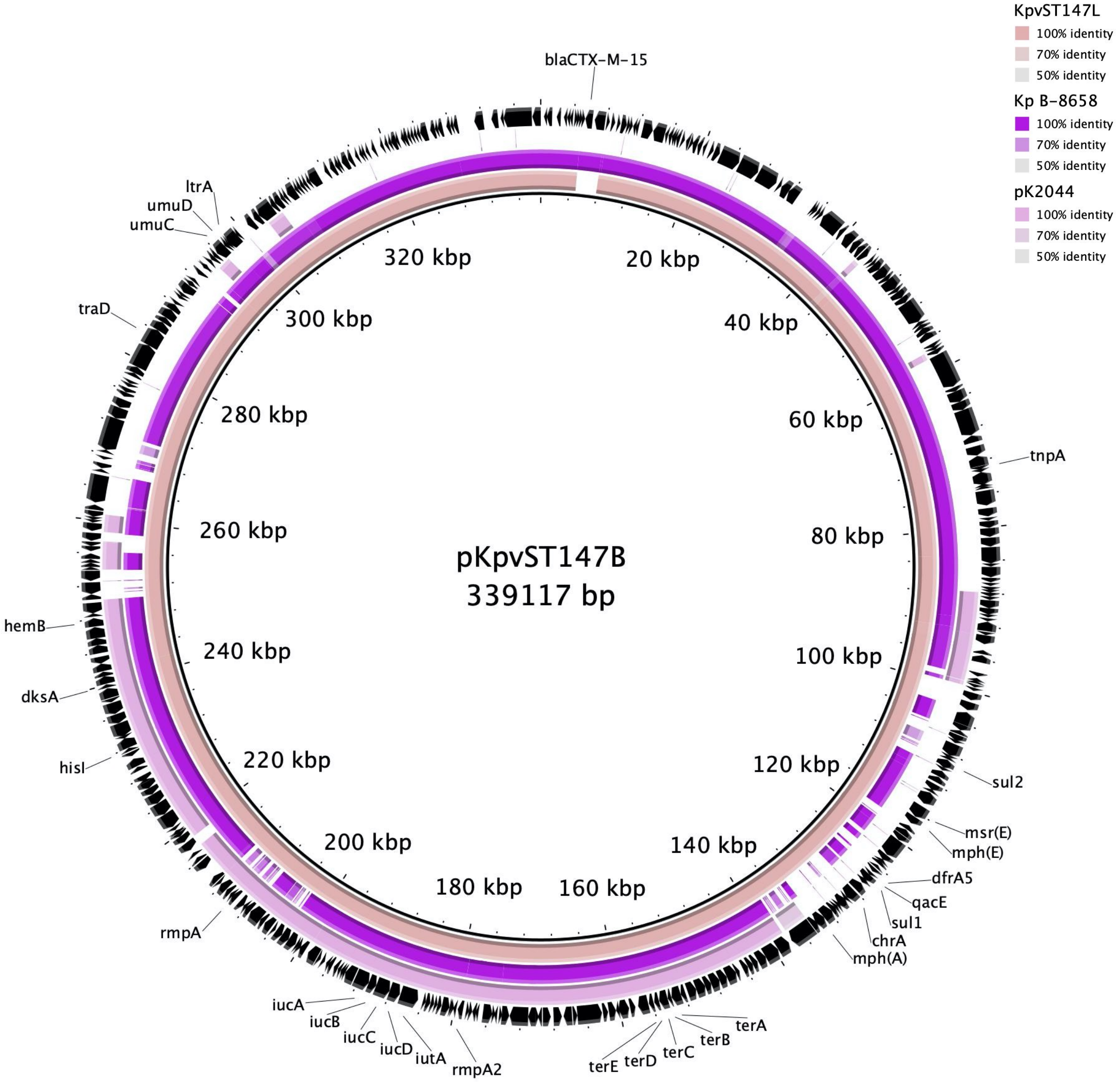

Figure S5. Sequence alignments of MDR-Hv plasmids identified in CG147 genomes.

Comparison of MDR-Hv plasmids identified in CG147 genomes (KpvST147L_NDM and B-8658) with the reference virulence plasmid pK2044 (AP006726.1) and a similar plasmid recovered in 2019 (pKpvST147B, CP040726.1), which was used as reference. The outermost circle shows annotations of the reference strain.

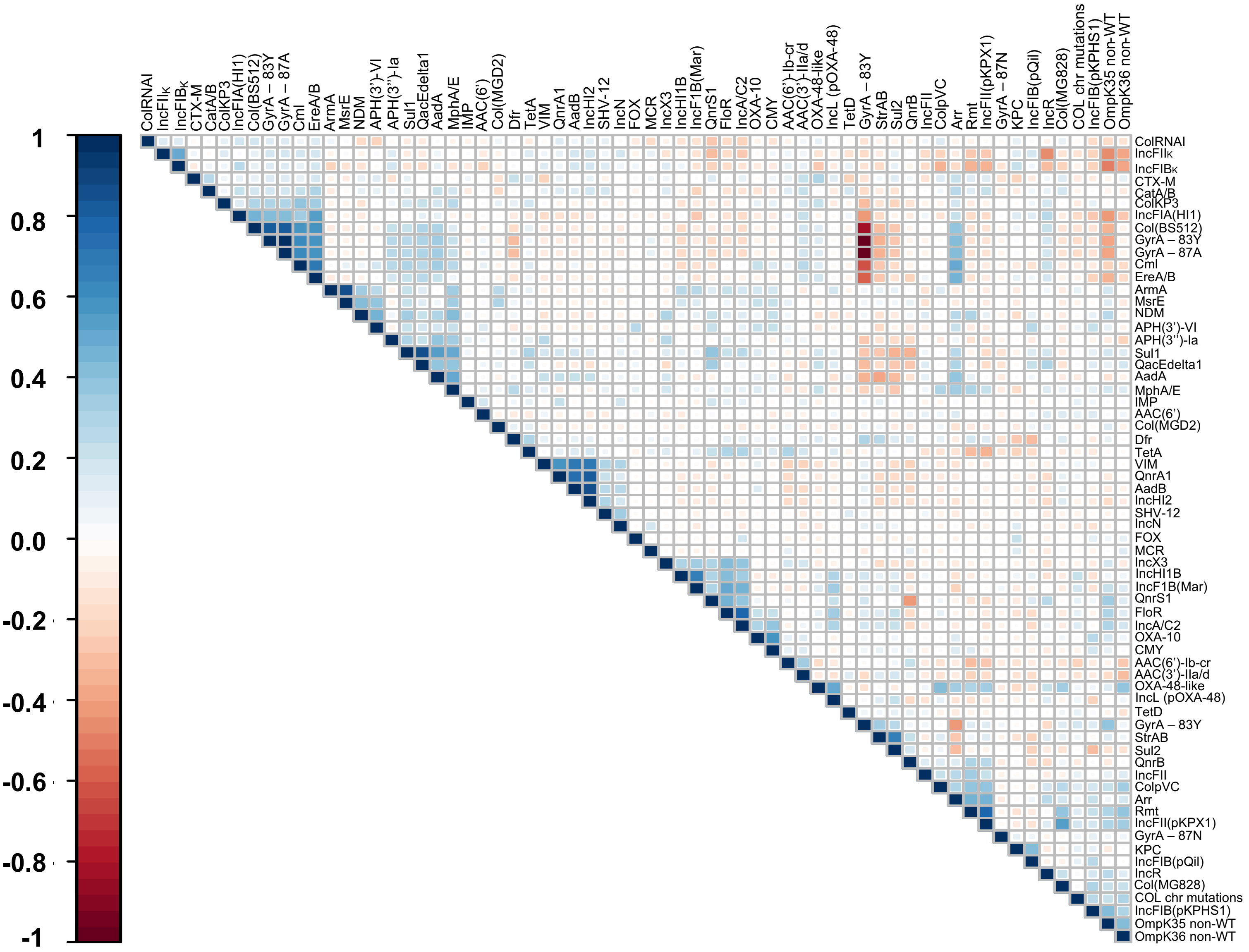

Figure S6. Correlation between genotypes (Pearson method).
Blank squares represent correlations without statistical significance (p > 0.05). The plot was created with the corrplot R package.

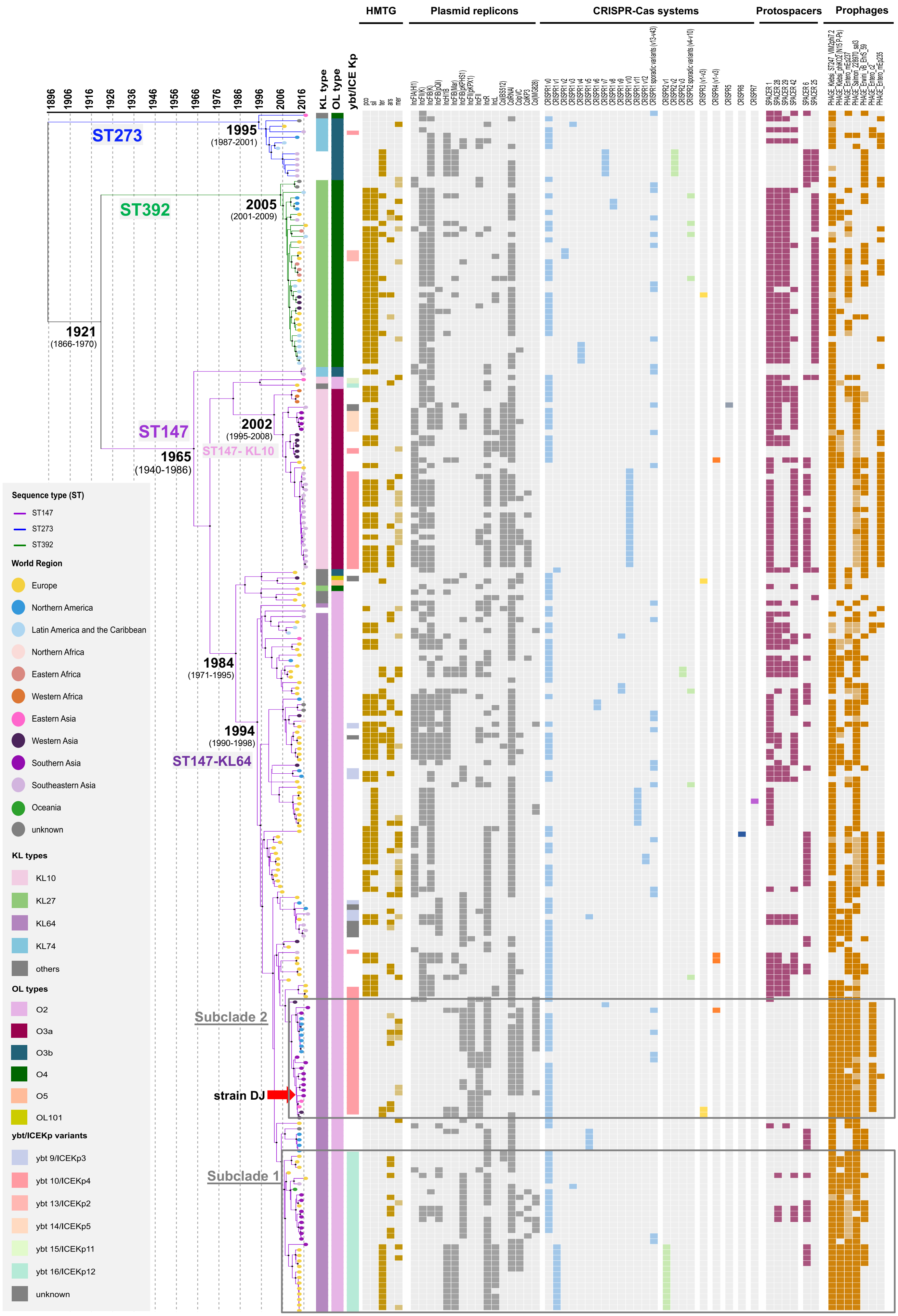

HMTG
Plasmid replicons
CRISPR-Cas systems
Protospacers
Prophages
KL type
OL type
ybt/ICE Kp
1896
1906
1916
1926
1936
1946
1956
1966
1976
1986
1996
2006
2016
ST273
1995
(1987-2001)
ST392
2005
(2001-2009)
1921
(1866-1970)
ST147
2002
(1995-2008)
ST147- KL10
1965
(1940-1986)
1984
(1971-1995)
1994
(1990-1998)
ST147-KL64
Subclade 2
strain DJ
Subclade 1
Sequence type (ST)
ST147
ST273
ST392
World Region
Europe
Northern America
Latin America and the Caribbean
Northern Africa
Eastern Africa
Western Africa
Eastern Asia
Western Asia
Southern Asia
Southeastern Asia
Oceania
unknown
KL types
KL10
KL27
KL64
KL74
others
OL types
O2
O3a
O3b
O4
O5
OL101
ybt/ICEKp variants
ybt 9/ICEKp3
ybt 10/ICEKp4
ybt 13/ICEKp2
ybt 14/ICEKp5
ybt 15/ICEKp11
ybt 16/ICEKp12
unknown

Figure S7. **Time-scaled phylogeny of CG147 genomes and their heavy metal tolerance genes, plasmid replicons, CRIPSR systems, and prophages profile.**

The phylogeny was obtained using the BEAST tool. The three main branches correspond to the three main sequence types (ST). Tree tips are colored by world region of isolation (see key). Black dots on main nodes indicate ≥95% posterior probability. The grey boxes delineate subclades 1 and 2, as indicated. Capsular (KL) and O-antigen (O) locus types and the yersiniabactin-carrying ICE*Kp* elements are colored according to their variants as shown in the legend. The presence of heavy metal tolerance genes, plasmid replicons, CRISPR-Cas systems, protospacers and prophages is indicated. In heavy metal tolerance genes columns, lighter yellow indicates an incomplete operon. In prophages lighter orange indicates an questionable prophage. HMTG, heavy metal tolerance genes.

Table S2. CRISPR/Cas systems and variants identified within CG147 *K. pneumoniae* genomes.

CRISPR system/variant	No. of genomes	Size (bp)	DR consensus	No. of spacers	Comments
**CRISPR1 v0**	122	2650	GTGTTCCCCGCGCCAGCGGGGATAAACCG	43	considered as reference
**CRISPR1 v1**	13	2162	GTGTTCCCCGCGCCAGCGGGGATAAACCG	35	missing spacers 11 to 18
**CRISPR1 v2**	2	2162	GTGTTCCCCGCGCCAGCGGGGATAAACCG	35	missing spacers 35 to 43
**CRISPR1 v3**	2	2528	GTGTTCCCCGCGCCAGCGGGGATAAACCG	41	missing spacers 2 and 3
**CRISPR1 v4**	4	2675	GTGTTCCCCGCGCCAGCGGGGATAAACCG	43	duplication in one DR
**CRISPR1 v5**	5	2589	GTGTTCCCCGCGCCAGCGGGGATAAACCG	42	missing spacer 3
**CRISPR1 v6**	2	2223	GTGTTCCCCGCGCCAGCGGGGATAAACCG	36	missing spacers 21 to 27
**CRISPR1 v7**	6	2406	GTGTTCCCCGCGCCAGCGGGGATAAACCG	39	missing spacers 21 to 24
**CRISPR1 v8**	2	2406	GTGTTCCCCGCGCCAGCGGGGATAAACCG	39	missing spacers 36 to 39
**CRISPR1 v9**	3	2528	GTGTTCCCCGCGCCAGCGGGGATAAACCG	41	missing spacers 38 and 39
**CRISPR1 v10**	18	1735	GTGTTCCCCGCGCCAGCGGGGATAAACCG	28	missing spacers 12 to 16; 21 to 29 and 38
**CRISPR1 v11**	7	1308	GTGTTCCCCGCGCCAGCGGGGATAAACCG	21	missing spacers 17 to 38
**CRISPR1 v12**	2	1369	GTGTTCCCCGCGCCAGCGGGGATAAACCG	22	missing spacers 9 to 29
**CRISPR1 v13**	1	2772	GTGTTCCCCGCGCCAGCGGGGATAAACCG	45	two extra spacers; spacer 26 different
**CRISPR1 v14**	1	2679	GTGTTCCCCGCGCCAGCGGGGATAAACCG	43	missing spacer 27; spacer 26 different; extra spacer
**CRISPR1 v15**	1	2650	GTGTTCCCCGCGCCAGCGGGGATAAACCG	43	missing spacer 38; extra spacer in the beginning
**CRISPR1 v16**	1	2616	GTGTTCCCCGCGCCAGCGGGGATAAACCG	42	missing spacer 3
**CRISPR1 v17**	1	2589	GTGTTCCCCGCGCCAGCGGGGATAAACCG	42	missing spacer 34
**CRISPR1 v18**	1	2589	GTGTTCCCCGCGCCAGCGGGGATAAACCG	42	missing spacer 25
**CRISPR1 v19**	1	2589	GTGTTCCCCGCGCCAGCGGGGATAAACCG	42	missing spacer 38
**CRISPR1 v20**	1	2558	GTGTTCCCCGCGCCAGCGGGGATAAACCG	41	missing spacers 5 and 6
**CRISPR1 v21**	1	2553	GTGTTCCCCGCGCCAGCGGGGATAAACCG	41	missing spacers 41 and 42: duplication in one DR
**CRISPR1 v22**	1	2528	GTGTTCCCCGCGCCAGCGGGGATAAACCG	41	missing spacers 26 and 27

CRISPR system/variant	No. of genomes	Size (bp)	DR consensus	No. of spacers	Comments
**CRISPR1 v23**	1	2528	GTGTTCCCCGCGCCAGCGGGGATAAACCG	41	missing spacers 41 and 42
**CRISPR1 v24**	1	2528	GTGTTCCCCGCGCCAGCGGGGATAAACCG	41	missing spacers 30 to 31
**CRISPR1 v25**	1	2528	GTGTTCCCCGCGCCAGCGGGGATAAACCG	41	missing spacers 11 to 12
**CRISPR1 v26**	1	2467	GTGTTCCCCGCGCCAGCGGGGATAAACCG	40	missing spacers 40 to 42
**CRISPR1 v27**	1	2467	GTGTTCCCCGCGCCAGCGGGGATAAACCG	40	missing spacers 26 to 28
**CRISPR1 v28**	1	2406	GTGTTCCCCGCGCCAGCGGGGATAAACCG	39	missing spacers 26 to 29
**CRISPR1 v29**	1	2406	GTGTTCCCCGCGCCAGCGGGGATAAACCG	39	missing spacers 24 to 27
**CRISPR1 v30**	1	2406	GTGTTCCCCGCGCCAGCGGGGATAAACCG	39	missing spacers 14-16 and 27
**CRISPR1 v31**	1	2344	GTGTTCCCCGCGCCAGCGGGGATAAACCG	38	missing spacers 1-5
**CRISPR1 v32**	1	2284	GTGTTCCCCGCGCCAGCGGGGATAAACCG	38	missing spacers 37 to 42
**CRISPR1 v33**	1	2223	GTGTTCCCCGCGCCAGCGGGGATAAACCG	36	missing spacers 17; 35 to 40
**CRISPR1 v34**	1	2187	GTGTTCCCCGCGCCAGCGGGGATAAACCG	35	missing spacers 8 and 9; 11 to 22
**CRISPR1 v35**	1	2100	GTGTTCCCCGCGCCAGCGGGGATAAACCG	34	missing spacers 21 to 29
**CRISPR1 v36**	1	2040	GTGTTCCCCGCGCCAGCGGGGATAAACCG	33	missing spacers 17; 31 to 33, 35 to 40
**CRISPR1 v37**	1	1887	GTGTTCCCCGCGCCAGCGGGGATAAACCG	30	missing spacers 20 to 32
**CRISPR1 v38**	1	1673	GTGTTCCCCGCGCCAGCGGGGATAAACCG	27	missing spacers 4, 5 and 18 to 31
**CRISPR1 v39**	1	1674	GTGTTCCCCGCGCCAGCGGGGATAAACCG	27	missing spacers 8 to 22, and 25
**CRISPR1 v40**	1	1399	GTGTTCCCCGCGCCAGCGGGGATAAACCG	22	missing spacers 13 to 33
**CRISPR1 v41**	1	1276	GTGTTCCCCGCGCCAGCGGGGATAAACCG	21	missing spacers 15 to 37
**CRISPR1 v42**	1	1003	GTGTTCCCCGCGCCAGCGGGGATAAACCG	16	missing spacers 8 to 14; and 16 to 35
**CRISPR1 v43**	1	575	GTGTTCCCCGCGCCAGCGGGGATAAACCG	9	missing spacers 4 to 20; 23 to 34 and 37 to 41
**CRISPR2 v1**	12	1056	CCGATAACCCCCGCATGCGGGGGAATAC	17	-
**CRISPR2 v2**	5	1549	CCGATAACCCCCGCATGCGGGGGAATAC	25	-

CRISPR system/variant	No. of genomes	Size (bp)	DR consensus	No. of spacers	Comments
**CRIPSR2 v3**	2	515	CCGATAACCCCCGCATGCGGGGGGAATAC	8	-
**CRISPR2 v4**	1	1367	CCGATAACCCCCGCATGCGGGGGGAATAC	22	-
**CRISPR2 v5**	1	1245	CCGATAACCCCCGCATGCGGGGGGAATAC	20	-
**CRISPR2 v6**	1	939	CCGATAACCCCCGCATGCGGGGGGAATAC	15	-
**CRISPR2 v7**	1	814	CCGATAACCCCCGCATGCGGGGGGAATAC	13	-
**CRISPR2 v8**	1	761	CCGATAACCCCCGCATGCGGGGGGAATAC	12	-
**CRISPR2 v9**	1	696	CCGATAACCCCCGCATGCGGGGGGAATAC	11	-
**CRISPR2 v10**	1	456	CCGATAACCCCCGCATGCGGGGGGAATAC	7	-
**CRISPR3 v1**	2	1060	CCGATAACCCCCGCACACGGGGGGAATAC	17	-
**CRISPR3 v2**	1	1004	CCGATAACCCCCGCACACGGGGGGAATAC	16	-
**CRISPR3 v3**	1	638	CCGATAACCCCCGCACACGGGGGGAATAC	10	-
**CRISPR4 v1**	2	1238	CCGATAACCCCCGCATGCGGGGG	19	-
**CRISPR4 v2**	1	815	CCGATAACCCCCGCATGCGGGGG	13	-
**CRISPR4 v3**	1	631	CCGATAACCCCCGCATGCGGGGG	10	-
**CRISPR5**	1	1788	CCGATAACCCCCGCAAGCGGGGGAATAC	29	-
**CRISPR6**	1	599	CCGCGCCGGTGGACGCCCCGGCCGCCGAACCGGTCGA CCCGCGCAAGGCGGCGG	5	-
**CRISPR7**	1	399	AGAAACACCCCCACGTGCGTGGGGAAGAC	5	-
